# Supplementary material for: Rhizobacteria Mitigate the Negative Effect of Aluminum on Pea Growth by Immobilizing the Toxicant and Modulating Root Exudation
Source: Plants (Basel). 2022 Sep 16;11(18):2416. doi: 10.3390/plants11182416 (PMC9503566; doi:10.3390/plants11182416)
Supplement: Supplementary file 1 [file plants-11-02416-s001.zip › plants-1844462-supplementary.pdf]

Table S1. Utilization of organic acids, amino acids and sugars by *Ps. fluorescens* SPB2137 as a sole source of carbon or nitrogen in batch culture.

**Organic acids and sugars**		**Amnio acids**		
**Substance**	**Utilization as C source**	**Substance**	**Utilization as N source**	**Utilization as C source**
t-Aconitic acid	±	ACC	++	-
Arabinose	+++	Alanine	+++	+++
Citric acid	+++	Arginine	++	++
Fructose	+++	Aspartic acid	+++	++
Fumaric acid	+++	Glycine	+++	+++
Glucose	+++	Glutamic acid	+	-
Lactic acid	+++	Glutamine	++	+++
Malic acid	++	Histidine	+++	+++
Maltose	±	Isoleucine	+++	
Melibiose	-	Leucine	++	++
Oxalic acid	++	Lysine	+++	++
Propionic acid	±	Methionine	++	-
Pyroglutamic acid	+++	Phenylalanine	++	++
Pyruvic fcid	+++	Proline	++	++
Ribose	++	Serene	++	-
Succinic acid	++	Threonine	+	-
Sucrose	+++	Tryptophan	+	-
Xylose	++	Tyrosine	++	++
		Valine	+	+

+++ abundant growth; ++ average growth; + weak growth; - lack of growth. All presented properties of *Ps. fluorescens* SPB2137 and its GFP tagged variant SPB2137gfp were similar (data for SPB2137gfp are not shown).

Table S2. The effect of Al and *Ps. fluorescens* SPB2137gfp on exudation of organic acids (μg g^{-1} root dry weight) by the studied pea genotypes.

Treatments	Organic acid ions				
	**Acetate**	**Citrate**	**Lactate**	**Pyroglutamate**	**Succinate**
			Pea genotype VIR1903		
-Al-Pf	262 ± 49 c	16 ± 4 a	227 ± 24 b	126 ± 24 c	259 ± 59 b
-Al+Pf	65 ± 13 a	nd	92 ± 22 a	21 ± 10 a	18 ± 2 a
+Al-Pf	702 ± 63 d	6392 ± 829 d	419 ± 32 cd	266 ± 46 de	1546 ± 159 e
+Al+Pf	389 ± 49 c	6286 ± 1266 cd	541 ± 49 d	150 ± 29 cd	368 ± 87 bc
			Pea genotype VIR8473		
-Al-Pf	148 ± 14 b	16 ± 1 a	185 ± 19 b	5 ± 1 a	269 ± 50 b
-Al+Pf	nd	6 ± 1 a	45 ± 4 a	nd	nd
+Al-Pf	896 ± 153 d	3127 ± 535 bc	351 ± 45 c	40 ± 8 a	1017 ± 114 d
+Al+Pf	nd	1818 ± 398 b	320 ± 59 c	nd	17 ± 7 a
			Pea genotype VIR7307		
-Al-Pf	152 ± 23 b	6 ± 2 a	61 ± 13 a	330 ± 73 e	164 ± 47 b
-Al+Pf	45 ± 20 a	nd	70 ± 26 a	nd	64 ± 11 a
+Al-Pf	445 ± 83 c	2429 ± 801 bc	206 ± 29 b	281 ± 47 e	656 ± 126 cd
+Al+Pf	144 ± 32 b	3881 ± 1154 c	88 ± 26 a	189 ± 27 d	158 ± 7 b
			Pea genotype VIR8353		
-Al-Pf	104 ± 16 ab	nd	105 ± 21 a	194 ± 23 d	699 ± 111 cd
-Al+Pf	64 ± 17 a	nd	69 ± 29 a	14 ± 13 a	264 ± 14 b
+Al-Pf	363 ± 72 c	3412 ± 892 bc	688 ± 128 d	116 ± 27 c	1008 ± 106 d
+Al+Pf	77 ± 13 a	3529 ± 742 bc	223 ± 49 b	59 ± 16 b	604 ± 150 c

Treatments: -Al-Pf — Al-untreated and uninoculated control plants, -Al+Pf — Al-untreated and inoculated plants, +Al-Pf — Al-treated and uninoculated plants, +Al+Pf — Al-treated and inoculated plants. Data are means ± SE. Different lowercase letters show significant differences between treatments (least significant difference test, $p < 0.05$, **n** =3). nd stands for not detected.

Table S3. The effect of Al and *Ps. fluorescens* SPB2137gfp on exudation of amino acids (μg g^{-1} root dry weight) by the studied pea genotypes.

**Treatments**	**Amino acids**								
	**Ala**	**Arg**	**Asp**	**Glu**	**Gly**	**His**	**Ile**	**Leu**	**Lys**
				Pea genotype VIR1903					
-Al-Pf	17,2 ± 1.7 b	1,4 ± 0,3 a	2,0 ± 0,2 a	6,7 ± 1.8 b	0,9 ± 0.5 ab	26 ± 4 a	7,7 ± 0.5 d	5,4 ± 0.6 cd	7,9 ± 1.8 c
-Al+Pf	8,6 ± 1.8 a	nd	0,6 ± 0,2 a	0,5 ± 0.1 a	3.3 ± 0.6 c	nd	nd	0,1 ± 0.1 a	nd
+Al-Pf	31,1 ± 3.6 cd	nd	19,5 ± 3,6 d	37,3 ± 5.2 c	5.9 ± 1.5 cd	395 ± 5 e	3,7 ± 0.3 c	4,1 ± 0.1 c	6,1 ± 1.0 bc
+Al+Pf	30,4 ± 5.6 bc	nd	25,0 ± 3,5 d	34,8 ± 6.4 c	1.2 ± 0.1 a	38 ± 4 ab	9,6 ± 1.5 d	10,9 ± 1.6 d	13,3 ± 3.7 d
				Pea genotype VIR8473					
-Al-Pf	6,7 ± 1.6 a	6,9 ± 1,9 b	2,0 ± 0,1 a	7,2 ± 1.4 b	4,4 ± 0.4 cd	4 ± 1 a A	2,1 ± 0.6 b	2,3 ± 0.8 a	2,9 ± 1.0 ab
-Al+Pf	6,0 ± 0.8 a	6,7 ± 3,9 b	2,2 ± 1,0 a	9,6 ± 5.0 b	2,0 ± 0.3 b	nd	0,5 ± 0.3 a	0,8 ± 0.3 a	1,5 ± 0.4 a
+Al-Pf	2,0 ± 0.2 a	10.6 ± 1,5 b	2,8 ± 0,1 a	1,2 ± 0.1 a	2,3 ± 0.6 b	nd	0,6 ± 0.2 a	0,9 ± 0.4 a	1,0 ± 0.4 a
+Al+Pf	43,1 ± 8.0 d	7,7 ± 0.4 b	44,6 ± 5,8 e	47,9 ± 4.9 c	6,3 ± 1.4 d	18 ± 5 a	3,8 ± 0.9 bc	7,3 ± 1.9 d	6,5 ± 0.7 bc
				Pea genotype VIR7307					
-Al-Pf	5,6 ± 2.3 a	5,6 ± 3,2 b	4,2 ± 1,2 ab	9,5 ± 2.9 b	nd	56 ± 16 b	1,2 ± 0.2 ab	1,0 ± 0.2 a	1,8 ± 0.4 a
-Al+Pf	23,4 ± 2.7 bc	15,0 ± 6,9 b	0,4 ± 0,1 a	1,3 ± 0.3 a	0,5 ± 0.1 a	5 ± 3 a	0,7 ± 0.2 a	1,1 ± 0.4 a	0,9 ± 0.3 a
+Al-Pf	2,1 ± 0.1 a	nd	2,7 ± 0,1 a	5,9 ± 0.3 b	nd	244 ± 13 d	1,2 ± 0.1 ab	1,3 ± 0.2 a	3,7 ± 0.3 ab
+Al+Pf	9,2 ± 3.6 a	nd	10,9 ± 1,4 c	12,5 ± 3.5 b	0,9 ± 0.1 a	17 ± 10 a	3,4 ± 1.6 bc	3,6 ± 1.8 bc	8,1 ± 0.6 c
				Pea genotype VIR8353					
-Al-Pf	1,4 ± 0.3 a	nd	3,7 ± 0,2 a	5,4 ± 0.2 a	0,2 ± 0.1 a	68 ± 4 b	0,6 ± 0.1 a	0,6 ± 0.1 a	2,9 ± 0.5 ab
-Al+Pf	22,6 ± 6.4 bc	nd	1,0 ± 0,3 a	4,1 ± 1.6 a	0,3 ± 0.2 a	38 ± 10 ab	1,4 ± 0.5 ab	1,9 ± 0.8 a	3,9 ± 1.3 ab
+Al-Pf	0,3 ± 0.1 a	nd	3,4 ± 0,4 a	5,3 ± 1.2 a	0,3 ± 0.1 a	165 ± 4 c	0,4 ± 0.1 a	0,8 ± 0.1 a	5,7 ± 1.4 bc
+Al+Pf	8,6 ± 1.3 a	nd	6,9 ± 0,6 b	8,7 ± 1.0 a	0,4 ± 0.1 a	145 ± 16 c	2,6 ± 0.6 b	3,6 ± 1.0 bc	7,6 ± 1.5 c

Treatments: -Al-Pf — Al-untreated and uninoculated control plants, -Al+Pf — Al-untreated and inoculated plants, +Al-Pf — Al-treated and uninoculated plants, +Al+Pf — Al-treated and inoculated plants. Data are means ± SE. Different lowercase letters show significant differences between treatments (least significant difference test, $p < 0.05$, **n** =3). nd stands for not detected.

Table S4. The effect of Al and *Ps. fluorescens* SPB2137gfp on exudation of sugars (µg g^{-1} root dry weight) by the studied pea genotypes.

**Treatments**	**Amino acids**								
	**Met**	**Orn**	**Phe**	**Pro**	**Thr**	**Ser**	**Trp**	**Tyr**	**Val**
					Pea genotype VIR1903				
-Al-Pf	nd	0,3 ± 0.1 a	4,2 ± 0.4 b	1,5 ± 0.2 a	44 ± 1 b	7 ± 1 a	1,2 ± 0.3 a	1,3 ± 0.5 b	11,8 ± 0.3 c
-Al+Pf	nd	0,1 ± 0.1 a	0.1 ± 0.1 a	1,3 ± 0.1 a	102 ± 11 de	nd	0,8 ± 0.1 a	nd	2,6 ± 1.4 ab
+Al-Pf	2,6 ± 0.7 c	1,9 ± 0.2 b	3,7 ± 0.3 b	46,6 ± 7.3 f	112 ± 20 de	23 ± 4 a	2,3 ± 0.8 a	1,6 ± 0.3 b	5,1 ± 0.5 b
+Al+Pf	1,5 ± 0.5 bc	2,7 ± 0.1 b	8,2 ± 1.9 c	26,5 ± 6.6 e	126 ± 6 de	156 ± 22 c	1,1 ± 0.2 a	3,0 ± 1.0 b	13,3 ± 2.6 c
					Pea genotype VIR8473				
-Al-Pf	0,5 ± 0.3 ab	3,1 ± 1.2 b	0,9 ± 0.3 a	1,2 ± 0.1 a	4 ± 1 a	25 ± 7 a	0,4 ± 0.1 a	nd	3,1 ± 0.5 ab
-Al+Pf	nd	0,7 ± 0.2 a	1,1 ± 0.5 a	2,9 ± 0.2 a	4 ± 1 a	8 4 a	0,9 ± 0.4 a	nd	3,3 ± 0.6 ab
+Al-Pf	nd	0,6 ± 0.2 a	0,6 ± 0.1 a	1,0 ± 0.4 a	53 ± 12 bc	4 ± 1 a	0,8 ± 0.1 a	nd	1,6 ± 0.3 a
+Al+Pf	3,2 ± 1.0 c	2,6 ± 0.6 b	8,1 ± 1.9 c	15,9 ± 2.0 d	117 ± 3 de	234 ± 36 d	9,8 ± 2.5 d	nd	5,0 ± 1.9 ab
					Pea genotype VIR7307				
-Al-Pf	0,3 ± 0.1 a	0,9 ± 0.1 a	1,2 ± 0.3 a	4,6 ± 1.3 ab	87 ± 4 d	25 ± 12 a	5,1 ± 1.4 c	nd	11,0 ± 2.8 c
-Al+Pf	1,1 ± 0.4 b	0,4 ± 0.1 a	0,5 ± 0.1 a	4,4 ± 0.8 ab	137 ± 25 e	2 ± 1 a	2,7 ± 1.1 abc	nd	nd
+Al-Pf	0,3 ± 0.1 a	1,1 ± 0.1 a	1,2 ± 0.1 a	5,2 ± 0.2 ab	114 ± 5 de	6 ± 1 a	1,6 ± 0.1 a	nd	1,7 ± 0.1 a
+Al+Pf	0,4 ± 0.1 a	5,8 ± 1.1 c	2,9 ± 1.2 b	13,9 ± 4.8 cd	100 ± 16 de	80 ± 35 b	10,1 ± 1.6 d	nd	6,6 ± 1.1 b
					Pea genotype VIR8353				
-Al-Pf	nd	1,0 ± 0.1 a	0,7 ± 0.1 a	4,1 ± 0.2 ab	74 ± 3 bcd	6 ± 1 a	0,5 ± 0.1 a	0,6 ± 0,1 a	2,3 ± 0.3 a
-Al+Pf	nd	6,8 ± 0.6 c	2,2 ± 0.7 b	8,0 ± 3.3 bcd	83 ± 24 cd	13 ± 1 a	5,2 ± 0.7 c	2,7 ± 0.8 b	2,5 ± 0.7 a
+Al-Pf	nd	1,4 ± 0.1 a	0,7 ± 0.2 a	5,2 ± 0.8 ab	133 ± 12 e	6 ± 1 a	0,7 ± 0.1 a	0,7 ± 0.1 a	2,6 ± 0.3 a
+Al+Pf	0,2 ± 0.1 a	6,5 ± 0.8 c	3,1 ± 1.0 b	6,2 ± 0.5 abc	124 ± 21 e	57 ± 4 b	3,7 ± 0.2 bc	3,0 ± 0.8 b	5,9 ± 0.7 b

: -Al-Pf — Al-untreated and uninoculated control plants, -Al+Pf — Al-untreated and inoculated plants, +Al-Pf — Al-treated and uninoculated plants, +Al+Pf — Al-treated and inoculated plants. Data are means ± SE. Different lowercase letters show significant differences between treatments (least significant difference test, $p < 0.05$, **n** =3). nd stands for not detected.

Table S5. The effect of Al and *Ps. fluorescens* SPB2137gfp on exudation of sugars (μg g^{-1} root dry weight) by the studied pea genotypes.

**Treatments**	**Sugars**		
	**Fructose**	**Glucose**	**Ribose**
	Pea genotype VIR1903		
-Al-Pf	5,8 ± 0,5 c	1,18 ± 0,21 bc	0,18 ± 0,05 b
-Al+Pf	0,1 ± 0,1 a	nd	nd
+Al-Pf	18,4 ± 1,9 d	3,87 ± 0,91 d	0,51 ± 0,05 c
+Al+Pf	4,1 ± 0,2 b	0,86 ± 0,36 b	0,50 ± 0,06 c
	Pea genotype VIR8473		
-Al-Pf	1,1 ± 0,1 ab	0,17 ± 0,02 a	nd
-Al+Pf	0,2 ± 0,1 a	0,09 ± 0,02 a	nd
+Al-Pf	2,9 ± 0,2 b	1,18 ± 0,21 b	nd
+Al+Pf	2,6 ± 0,3 b	1,71 ± 0,22 bc	nd
	Pea genotype VIR7307		
-Al-Pf	6,8 ± 1,2 c	1,02 ± 0,04 b	0,13 ± 0,01 a
-Al+Pf	0,4 ± 0,2 a	0,25 ± 0,04 a	nd
+Al-Pf	20,9 ± 1,0 d	2,05 ± 0,37 c	0,20 ± 0,01 ab
+Al+Pf	18,1 ± 2,1 d	1,05 ± 0,06 b	0,10 ± 0,01 a
	Pea genotype VIR8353		
-Al-Pf	3,0 ± 0,6 b	0,13 ± 0,03 a	0,13 ± 0,01 a
-Al+Pf	0,5 ± 0,2 a	0,02 ± 0,01 a	nd
+Al-Pf	4,3 ± 0,3 b	0,17 ± 0,03 a	0,47 ± 0,06 c
+Al+Pf	1,9 ± 0,3 ab	0,08 ± 0,01 a	0,29 ± 0,05 b

Treatments: -Al-Pf — Al-untreated and uninoculated control plants, -Al+Pf — Al-untreated and inoculated plants, +Al-Pf — Al-treated and uninoculated plants, +Al+Pf — Al-treated and inoculated plants. Data are means ± SE. Different lowercase letters show significant differences between treatments (least significant difference test, $p < 0.05$, **n** =3). nd stands for not detected.

Table S6. Values of Fisher's criterion (F) assessing significance of the effects of factors and their interactions on the studied parameters

Factors and their interactions	Parameters															
	Pea biomass			Number of bacteria		Al concentrations				Solution pH	P concentrations			Root exudation		
	Root	Shoots	Whole plant	Solution	Roots	Solution	Residue	Roots	Shoots		Residue	Roots	Shoots	Organic acids	Amino acids	Sugars
Pea	77 ***	16 ***	57 ***	4 *	27 ***	4 *	15 ***	34 ***	18 ***	61 ***	16 ***	99 ***	42 ***	13 ***	10 ***	132 ***
Al	12 **	48 ***	37 ***	143***	206 ***	ndt	ndt	ndt	ndt	113 ***	93 ***	42 ***	1	262 ***	208 ***	348 ***
Pf	3	9 **	10 **	ndt	ndt	25 ***	67 ***	19 ***	1	41 ***	119 ***	1	1	11 **	2	144 ***
Pea × Al	2	4 *	4 *	7 ***	22 ***	ndt	ndt	ndt	ndt	3	11 ***	3	1	12 **	17 ***	64 ***
Pea × Pf	2	3 *	2	ndt	ndt	3	6 *	1	< 1	2	13 ***	1	1	2	16 ***	35 ***
Al × Pf	2	5 *	4	ndt	ndt	ndt	ndt	ndt	ndt	8 **	14 ***	2	< 1	2	3	3
Pea × Al × Pf	0,9	3	2	ndt	ndt	ndt	ndt	ndt	ndt	2	4 *	1	< 1	2	18 ***	12 ***

Factors: Pea — pea genotype; Al — treatment with Al; Pf — Inoculation with bacteria. F values marked by asterisks show significant effects (*— $p < 0.05$; ** — $p < 0.01$; *** — $p < 0.001$). ndt stands for not determined.
